# IntegriLAB: a blockchain-enabled electronic lab notebook for reproducible neuroimaging research

**DOI:** 10.3389/fninf.2026.1753937

**Published:** 2026-05-21

**Authors:** Rubaida Easmin, Mattia Veronese, Paul Allen, Giovanna Nordio, Federico Turkheimer

**Affiliations:** 1 Institute of Psychiatry, Psychology and Neuroscience, King’s College London, London, United Kingdom; 2 Department of Information Engineering, University of Padua, Padua, Italy; 3 WILL Chair PSY Team, Centre LilNCog, INSERM U-1172, Lille, France

**Keywords:** blockchain verification, data integrity, electronic lab notebook, neuroimaging, research reproducibility

## Abstract

In the era of data-intensive science, managing and verifying research processes that include raw data, analysis scripts, workflows, and documentation, remains a major challenge, particularly in complex fields like neuroimaging were multiple processing and analyses stages can take place. Fragmented tools, inconsistent documentation, and poor platform integration continue to undermine reproducibility. While current Electronic Lab Notebooks (ELNs) aid organization, they often lack cross-platform interoperability, authorship certification, and immutable auditability. To address these gaps, this study proposes a novel ELN framework IntegriLAB, that consolidates traditional data, document and code repositories into a centralized, web-based system for end-to-end project tracking. As a proof of concept, our case study integrates DataLad, Overleaf and GitHub. It allows real-time monitoring across the research cycle while preserving familiar workflows. A key innovation is the use of LabTrace built on green blockchain technology to certify research activities through cryptographically signed, immutable records, ensuring data integrity and verifiable authorship with minimal environmental impact. By unifying project management, secure data handling, and blockchain-based verification, the proposed ELN advances reproducibility, fosters trust, and strengthens collaborative research practices.

## 1 Introduction

Technology and digitalization have dramatically altered research, moving it toward faster, automated, collaborative, and data-driven methodologies. However, scientists now face significant challenges in managing the vast volume of research data and associated files produced throughout scientific studies. These files include scripts, tests, descriptions of scientific workflows, variables from experimental data, execution environments (both software and hardware), manuscript files and other related materials (Wittek et al., 2021).

Neuroimaging studies are no exception to this trend. These studies are particularly notable for their intricate workflows, which include multiple stages such as data collection, data preparation, experimental setup, analysis execution and statistical thresholding, documentation, and publication of findings. The complexity of managing these resources is increased by the use of multiple data sources and a variety of data formats and contents. Although several standards and tools have been developed to improve aspects of reproducibility, managing and tracking the entire research workflow remains challenging. For example, the Brain Imaging Data Structure (BIDS) (Gorgolewski et al., 2016) standard facilitates the organization of neuroimaging datasets and metadata, while platforms such as NeuroDesk provide container-based environments that improve the portability and reproducibility of analysis pipelines (Renton et al., 2024). Nevertheless, the complexity and high degree of operations involved in neuroimaging workflows still makes it difficult to effectively track and manage all research activities while ensuring reproducibility of results (Niso et al., 2022; Borghi and Van Gulick, 2018; Lindquist, 2020).

Common scientific data management practices largely focus on archiving and disseminating final research outputs including processed datasets, publications and figures. These platforms emphasize how and where data should be stored and indexed after research is completed. However, to ensure published studies are reproducible, it is essential to record every detail of the active research workflow, showing how findings generalize across different processing methods or sample populations (Kennedy et al., 2019). Despite this need, there remains a critical gap in systems that support active, ongoing research workflows. Researchers lack tools that can continuously and immutably record the entire data lifecycle from the acquisition of raw data, through the transformations and analyses applied to the generation of final results ensuring full traceability and transparency throughout the research process (Bell et al., 2017).

A solution to this challenge is the implementation of Electronic Lab Notebook (ELN). An ELN in the context of neuroimaging research is a digital platform designed to document, organize, and manage every stage of a neuroimaging project from data acquisition and preprocessing to analysis, visualization, and publication (Kanza et al., 2017). Examples of widely used ELNs include LabArchives,^1^ LabInform (Schröder and Biskup, 2023), SciNote,^2^ eLabFTW (CARPi et al., 2017), Benchling,^3^ eLabNext,^4^ and Genemod.^5^ These platforms are designed to support the management of the datasets, inventory, project documentation, and collaborative workflows. While open-source systems like LabInform and eLabFTW require self-hosting and technical expertise (Schröder and Biskup, 2023; CARPi et al., 2017), commercial platforms such as LabArchives and Benchling offer subscription-based cloud services (Wittek et al., 2021; Gorgolewski et al., 2016; Renton et al., 2024).

Despite their advantages, integrating ELNs into research workflows poses several challenges particularly in fields that generate large and evolving datasets. Continuous uploading and monitoring of such data can be impractical. Some platforms, like LabArchives, allow users to link to externally stored data; however, this introduces complexity, as users must maintain link validity, ensure long-term accessibility, and manually update paths when files are renamed, moved, or deleted. Moreover, while certain systems automatically track revisions of internal files, they do not preserve version histories for externally stored data, leaving users responsible for manual documentation. This reliance on consistent file naming and storage conventions increases the risk of errors, broken references, and inefficient data retrieval, ultimately making long-term data integrity difficult to sustain.

Another major limitation of current ELNs is their limited integration with analytical workflows applied to research datasets. Most ELNs are designed primarily for documentation, offering minimal support for executing or tracking computational analyses. Researchers must often upload scripts and workflows manually, making it difficult to synchronize datasets with their corresponding analytical code or software environments. Features such as automated workflow tracking, metadata inheritance, and direct integration with Git-annex repositories are typically absent, hindering reproducibility of reported results.

Furthermore, although many ELNs provide secure audit trails and access logs, they typically rely on centralized databases controlled by vendors or institutions. In principle, administrators with sufficient privileges can modify or delete records, and database migrations may inadvertently alter timestamps or histories. Without an independent verification mechanism, ELNs cannot provide conclusive proof that a record existed at a specific time limiting their reliability for regulatory compliance, transparency, intellectual property protection, and reproducibility assurance.

Several existing tools already address individual aspects of these issues. For example, DataLad (Szczepanik et al., 2024) facilitates dataset versioning and provenance tracking; GitHub^6^ supports collaborative development and version control across analytical pipelines, independent of programing language or software environment; and Overleaf^7^ streamlines scientific documentation and publication writing. However, duplicating these functionalities within a single ELN would be redundant. What remains lacking, however, is a unified generalizable framework that integrates these tools into a single environment, allowing users to visualize and manage every stage of a research project cohesively.

To address this gap, this study introduces IntegriLAB, an integrated ELN framework that unifies DataLad, GitHub, and Overleaf within a single platform for managing data, source code, and documentation. IntegriLAB enables researchers to continue using familiar tools while benefiting from centralized project coordination and traceability. The system logs all research activities from data collection and preprocessing to analysis and reporting reducing workflow fragmentation and ensuring accessibility of project updates within one environment.

A defining feature of IntegriLAB is its comprehensive project logging system, which automatically records dataset and code modifications, updates, and milestones using Git-based version control. This automation minimizes human error and enhances transparency, enabling researchers, collaborators, and reviewers to trace every stage of a study, thereby strengthening reproducibility and accountability. In addition, IntegriLAB incorporates LabTrace, a blockchain-based verification module built on the Algorand network.^8^ LabTrace secures key research stages through cryptographic timestamps and digital signatures, ensuring data integrity, provenance, and immutable audit trails. This blockchain layer provides verifiable proof of authorship and ownership while maintaining a low environmental footprint through Algorand’s energy-efficient consensus protocol.

The remainder of this manuscript is organized as follows. Section 2 provides background context, outlining the major challenges in neuroimaging research, the shortcomings of existing workflow management solutions, and the potential of blockchain technology to address these issues. Section 3 describes the architecture, functionalities, and implementation of IntegriLAB, emphasizing its data privacy and transparency features. Section 4 presents a real-world case study demonstrating the system’s application in a neuroimaging research setting. Section 5 discusses the implications and benefits of the proposed approach, and section 6 concludes with future research directions.

## 2 Background and motivation

In today’s evolving research landscape, there is an increasing emphasis on openness, collaboration, and the seamless integration of tools, technologies, and administrative workflows.^9^ The rapid expansion of digital solutions has given rise to a wide array of applications designed to streamline various aspects of the research lifecycle. This section explores the key challenges researchers face and reviews existing tools, laying the groundwork for the introduction of IntegriLAB, the motivation behind its development and the needs it aims to address.

### 2.1 Challenges in reproducibility of neuroimaging research

**FIGURE 1:**
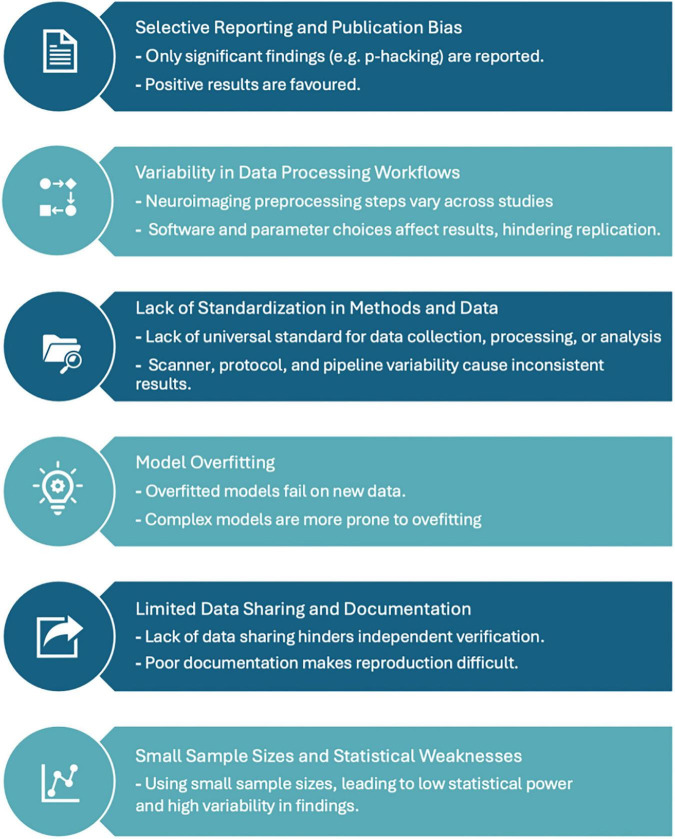
Graphical overview of challenges in reproducibility of neuroimaging research.

Reproducibility, defined as the ability to obtain consistent results using the same data and code (National Academies of Sciences; Engineering, and Medicine et al., 2019), remains a cornerstone of scientific integrity. In neuroimaging research, for example, reproducibility challenges persist, undermining the credibility and reliability of findings (Gorgolewski and Poldrack, 2016; Baker, 2016; Botvinik-Nezer et al., 2020; Button et al., 2013). As illustrated in Figure 1, these issues span multiple stages of the neuroimaging workflow including data acquisition, preprocessing, statistical analysis, visualization, and dissemination. Each stage is characterized by high data complexity, multiple dependencies, and domain-specific tools, contributing to variability across laboratories and studies.

A significant barrier to reproducibility is the absence of standardized protocols for conducting and analyzing neuroimaging experiments (Gorgolewski and Poldrack, 2016). Differences in scanner hardware, acquisition parameters, preprocessing pipelines, and statistical methodologies often result in divergent outcomes, even when analyzing similar datasets (Baker, 2016; Botvinik-Nezer et al., 2020). Moreover, many studies fail to comprehensively share scripts, metadata, or computational environments, making it difficult to independently verify or replicate results (Botvinik-Nezer et al., 2020).

The complexity of neuroimaging workflows also leads to fragmented data management practices (Loreggia et al., 2025). Research files are commonly dispersed across cloud services, local servers, and external repositories, often stored with inconsistent naming conventions, directory structures, and metadata standards. These inefficiencies hinder version control, compromise data provenance, and introduce avoidable redundancy (Zhao et al., 2023). In collaborative settings, particularly in multi-institutional or interdisciplinary projects, ambiguities in authorship and data ownership can further complicate accountability and raise concerns about intellectual property rights (White et al., 2022).

### 2.2 ELNs: a step toward reproducibility

In response to these challenges, ELNs have emerged as digital replacements for traditional paper records, offering structured, searchable, and collaborative platforms for documenting research workflows (Riley et al., 2017; Higgins et al., 2022). Popular ELNs, such as LabArchives, SciNote, Benchling, Genemod, and open-source alternatives like eLabFTW and LabInform, provide features including project documentation, version control, collaborative access, API integrations, and support for structured data entry. In terms of deployment, some platforms are exclusively cloud-based, while others support local installation, enabling institutions to store data on their own infrastructure for greater control and privacy.

Although ELNs offer clear benefits, most of them are not tailored to the demands of high-throughput, computationally intensive domains such as neuroimaging. These platforms typically lack native support for domain-specific software (e.g., FreeSurfer, FSL, SPM) and programing environments such as MATLAB, Python, or R, forcing researchers to manage workflows and documentation across multiple systems. This fragmentation often leads to inconsistencies between datasets, analytical code, and reported results. Furthermore, commercial ELNs frequently impose restrictive data storage limits, for instance, LabArchives’ professional edition caps usage at 100 GB which is insufficient for the large datasets common in neuroimaging.^10^

As datasets grow in size, they are often stored on external servers, and ELNs typically maintain only the reference links or storage locations to inform users about their availability. However, maintaining these external links and version histories usually requires manual effort, increasing the likelihood of errors, broken connections, data loss, and reduced data integrity. While these ELNs can track changes to individual documents or files, they rarely provide cryptographically verifiable audit trails or immutable certifications of authorship and data provenance features that are increasingly essential for promoting transparency and trust in collaborative research. To illustrate these limitations, Table 1 compares commonly used ELNs in terms of hosting model, API integrations, workflow automation, and suitability for reproducible neuroimaging research.

**TABLE 1:** Comparison of commonly used electronic laboratory notebooks (ELNs).

ELN platform	Open source	Hosting model	Blockchain support	API/integration with open-source tools	Degree of manual intervention	Provenance and verification mechanism	Notes on reproducibility and neuroimaging suitability
LabArchives	No	Cloud-based (subscription)	No	Limited API; external datasets linked manually	High	Internal version history only	Focuses on documentation and storage; limited support for computational workflows and large datasets (1)
SciNote	Yes	Cloud-based or on-premise	No	REST API available; limited workflow automation	Moderate	Internal logging and metadata tracking	Supports collaboration and metadata annotation, but lacks deep integration with analytical pipelines (2)
Benchling	No	Cloud-based (subscription)	No	API available; optimized for wet-lab data	Moderate	Internal audit trails	Designed primarily for molecular biology; limited suitability for imaging workflows (3)
eLabFTW	Yes	Self-hosted	No	REST API available; requires custom integration	Moderate	File version tracking and metadata records	Flexible and open-source, but requires technical expertise and manual workflow coordination (CARPi et al., 2017)
LabInform	Yes	Self-hosted	No	Limited API; relies on user-defined tooling	High	Metadata-based provenance	Strong emphasis on metadata and FAIR principles, but steep setup and maintenance overhead (5)
Genemod	No	Cloud-based	No	API available; biology-focused integrations	Moderate	Internal activity logs	Optimized for genetic and biological data; not tailored to neuroimaging or computational pipelines (Borghi and Van Gulick, 2018)

### 2.3 Advancing data integrity with blockchain technology

Blockchain technology provides a framework to address many of the limitations in current ELNs. By functioning as a distributed ledger, blockchain records transactions in cryptographically linked blocks maintained across a decentralized peer-to-peer network. Each node validates transactions through a consensus algorithm, ensuring data integrity and immutability once appended to the chain. These core attributes, immutability, transparency, decentralization, and programmability make blockchain a robust platform for verifiable data management. Integration into scientific workflows can enhance provenance tracking, reproducibility, and the security of experimental data.^11^

Traditional blockchains, particularly those using Proof-of-Work (PoW), are energy-intensive, raising environmental and scalability concerns (Alzoubi and Mishra, 2023). To address this, green blockchain protocols, such as Algorand’s Pure Proof-of-Stake (PPoS), offer energy-efficient consensus mechanisms that maintain security and decentralization while minimizing environmental impact (consume only ∼0.000008 kWh per transaction).^12^,^13^ LabTrace, built on Algorand, is an independent platform developed and maintained by the affiliated research group. It provides an immutable, cryptographically verifiable system for authenticating and tracing scientific data, ideas, and digital assets.

While conventional version control systems and hash functions can track changes and verify data integrity, they lack the ability to ensure transparency, immutability, and decentralized trust. For example, Git resembles a digital ledger, but its history can be altered, transactions duplicated, and provenance lost, providing no guarantee of a single, tamper-proof source of truth (Just et al., 2016). Besides, it is optimized for text-based source code but are not designed to efficiently manage large or binary files commonly used in neuroimaging research (e.g., DICOM, NIfTI formats).^14^ Similarly, hash functions confirm data integrity but do not inherently prevent tampering or create verifiable audit trails once data leaves a controlled environment. LabTrace overcomes these limitations by combining cryptographic signatures, distributed validation, and consensus protocols on a public blockchain, producing tamper-resistant, auditable, and trustless digital records.

### 2.4 Toward an integrated solution: IntegriLAB

Building on these insights, we developed IntegriLAB, an integrated digital lab platform for reproducible, transparent neuroimaging research. While existing ELNs (Table 1) provide documentation, version tracking, and script management, they often operate on isolated components of the research workflow and require substantial manual effort to maintain consistency between datasets, analytical code, and documentation. IntegriLAB addresses these limitations by connecting existing tools that support core research workflows, automatically collecting activity logs, and maintaining a unified record linking datasets, code, and documentation throughout the research lifecycle. By centralizing provenance information and reducing manual tracking, the platform supports scalable collaboration while preserving established research practices. Because the system primarily manages metadata rather than full datasets, it can efficiently support projects of varying size without significant performance overhead. In addition, blockchain-based certification through LabTrace provides tamper-resistant verification of key research outputs, creating secure and persistent records of research activities and authorship. Together, these capabilities provide a structured framework for improving transparency and reproducibility in scientific research. The following sections describe the system architecture, implementation, and a case study demonstrating its application in a real-world neuroimaging project.

## 3 IntegriLAB overview

**FIGURE 2:**
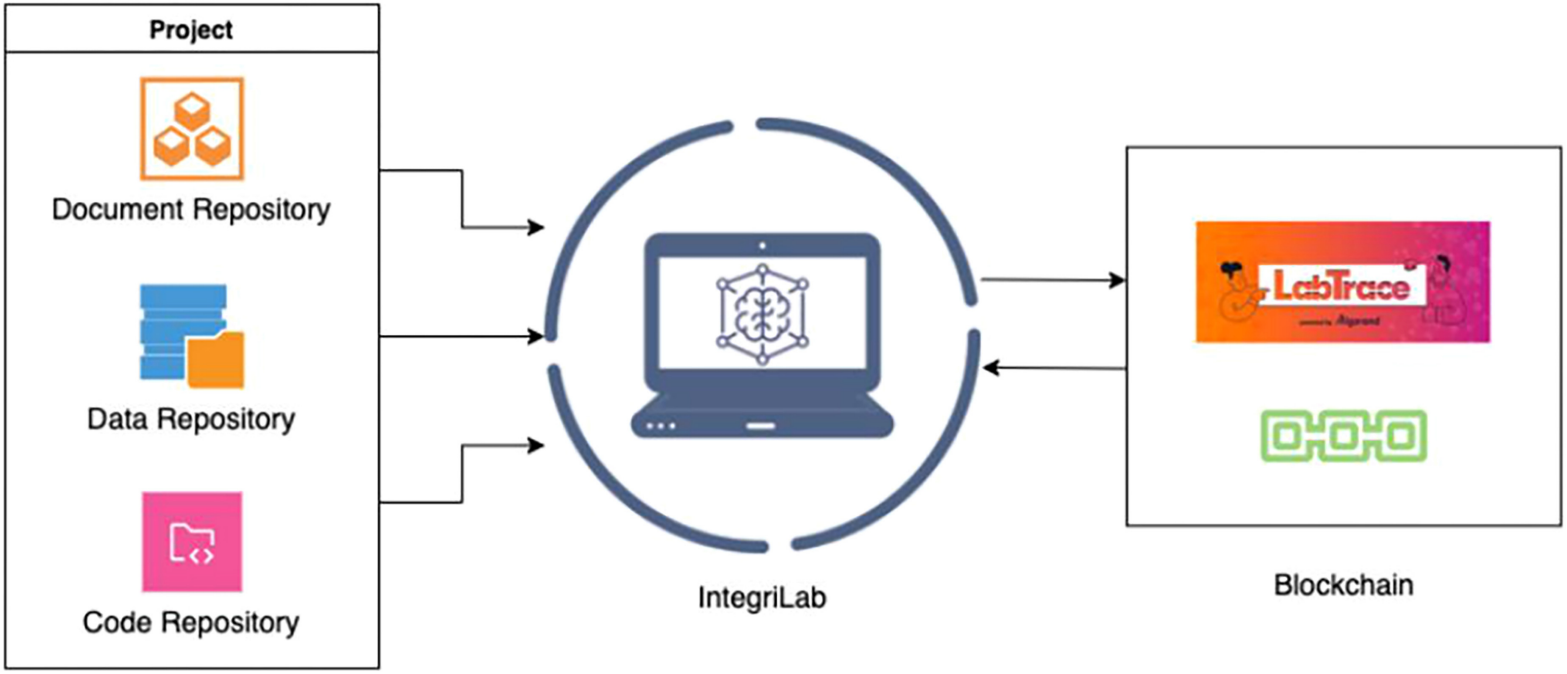
Overview of IntegriLAB.

IntegriLAB is a web-based application designed to support and streamline the management of research projects. The platform offers a consolidated framework by integrating several widely used open-source tools (Figure 2): Overleaf for collaborative manuscript writing, DataLad for data provenance and version control, and GitHub for source code management. This integration ensures that documentation, dataset histories, and analytical codes are consistently linked and traceable throughout the entire research lifecycle. Additionally, IntegriLAB connects with LabTrace that allows researchers to certify their work, providing tamper-proof validation of contributions. It is designed to flexibly document and track all research activities, independent of the specific experimental design or analysis pipeline. This makes the system well-suited for exploratory research environments, where workflows often evolve, as well as for structured settings, including large-scale collaborative studies and clinical research projects. The initial release of the application is accessible via:https://www.integrilab.org/.

### 3.1 System architecture

**FIGURE 3:**
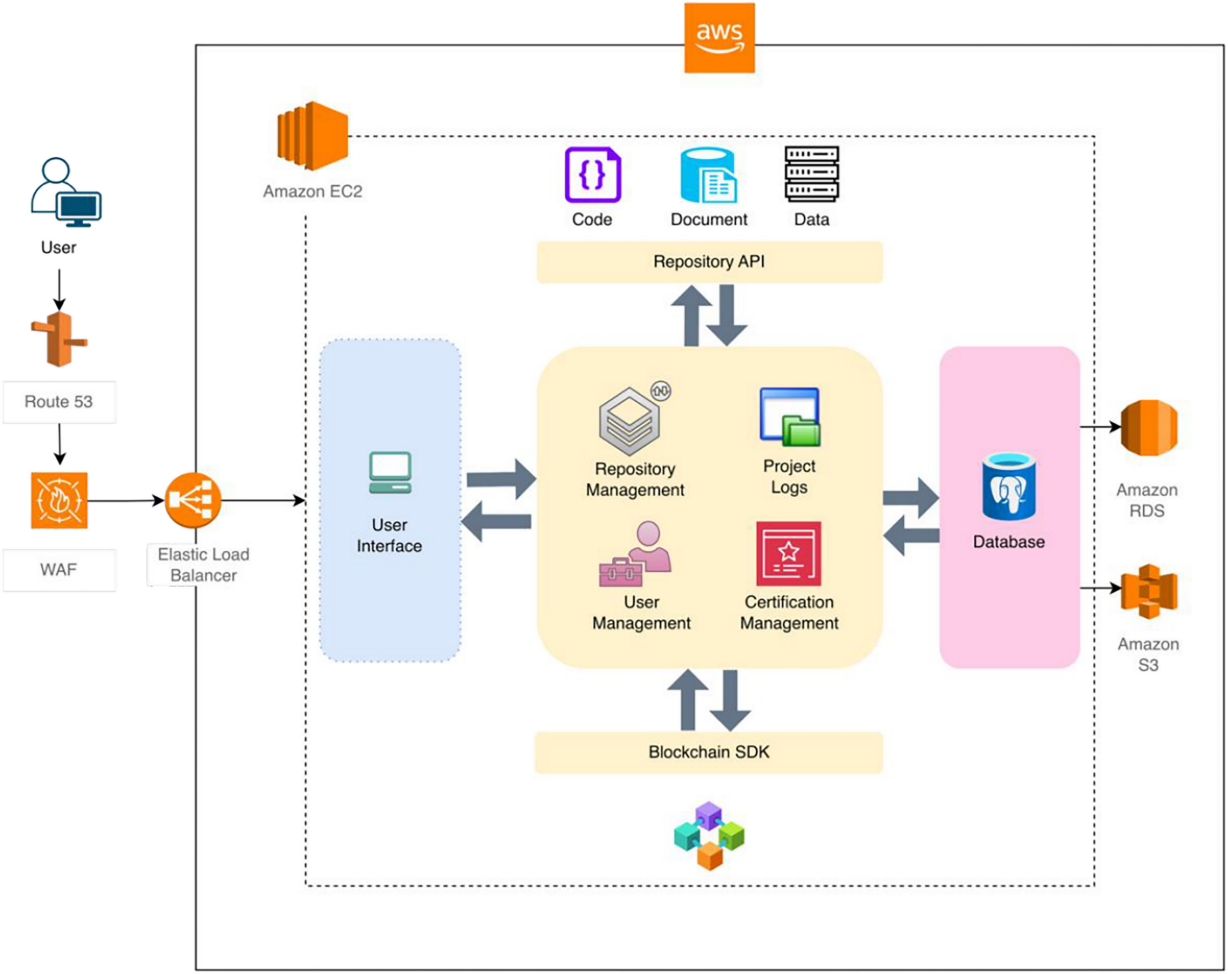
IntegrityLAB architecture. The dotted-line diagram illustrates the architecture of IntegriLAB, highlighting the frontend (light blue), backend (yellow), and database server (gray). The solid-line diagram shows the components involved in deploying the application on an AWS server.

This system follows a three-tier architecture, comprising a frontend user interface, a backend application layer, and a database, as illustrated in the dotted-line diagram in Figure 3. The frontend offers a user-friendly interface that manages user interactions and sends requests to the backend through RESTful APIs. This interface allows project members and administrators to interact with the system. Users can create and manage projects, connect profiles, track project progress, and engage with repositories.

The backend infrastructure is designed to efficiently process user requests, manage authentication, and oversee data storage. It plays a crucial role in user authentication, project management, and the integration of external services as well as in project certification. The backend communicates directly with the database to facilitate the storage of user and project data. Integration with external repositories is accomplished through the use of custom-developed APIs and open-source solutions for Overleaf (documents), DataLad (data), and GitHub (code). Furthermore, a custom-built SDK is employed to establish a connection with the blockchain platform LabTrace.

The database comprehensively stores all relevant information regarding users, projects, profiles, repository details, and project activities. It effectively manages roles and permissions for different user categories, including Administrator, Principal Investigator (PI), and general Member.

### 3.2 Workflow

**FIGURE 4:**
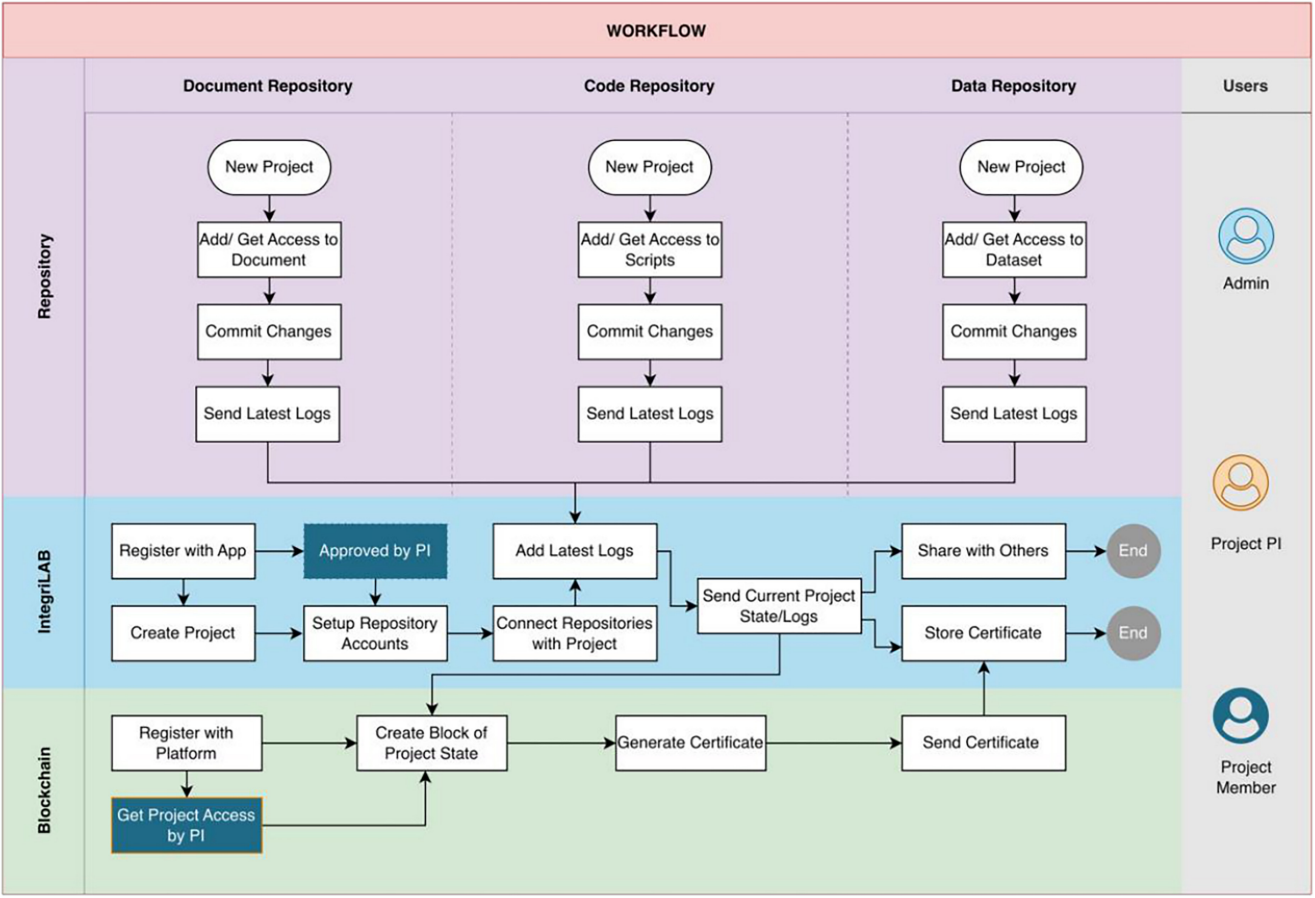
IntegrityLAB workflow. Principal Investigators (PIs) and Project Members follow a similar process for repository setup. However, Project Members must first be granted access by the PI within both the Blockchain and IntegriLAB platforms- highlighted in dark blue. The overall workflow is overseen by the Admin, whose responsibilities are shown in the sky-blue lane.

The workflow procedure for IntegriLAB is articulated in Figure 4. To access the application, users are required to complete a registration process. It is important for users to maintain active accounts on both Overleaf and GitHub, in addition to having a local or remote installation of DataLad for efficient data management. IntegriLAB accommodates three primary user roles: Administrator (responsible for user role Assignment and Entity management), Project Principal Investigator (PI, responsible for project setup and manage user access and permissions), and Project Member (responsible for log into project repository and blockchain validation).

The workflow is initiated by the PI, who is tasked with creating a new project, registering it with LabTrace for blockchain certification, and approving project members upon their registration. Following approval, project members must link their Overleaf, GitHub, and DataLad accounts to IntegriLAB and specify their respective repository names to ensure smooth integration across all tools.

Upon completion of the initial setup, users are able to commit updates from their connected repositories, which may include documentation, datasets, and source code, directly through IntegriLAB. This integration logs details from each repository, thereby promoting a transparent and traceable research process. At any point in the project timeline, users have the option to initiate project certification via LabTrace, which produces tamper-proof digital records that affirm authorship and ownership of research contributions.

### 3.3 Implementation and deployment

The IntegriLAB platform is deployed as a web-based application accessible via any modern browser, requiring an internet connection and user registration for access. Table 2 outlines the complete technology stack employed in its development. The system architecture emphasizes modularity, robustness, and extensibility, enabling integration of external research tools through dedicated APIs. In its current implementation, IntegriLAB integrates commonly used platforms including Overleaf, GitHub, DataLad, and the blockchain certification service LabTrace. The modular architecture allows additional services to be integrated without modifying the core platform. New integrations can be implemented through custom API connectors, enabling the framework to incorporate emerging tools such as container-based environments (e.g., Docker) or additional document management services. Once integrated, these services can be accessed by users directly through the platform interface without requiring advanced technical configuration.

**TABLE 2:** Comprehensive technology stack used for developing IntegriLAB.

Component	Technology/tool
Frontend	Bootstrap, JavaScript
Backend	Python (3.12), Django (5.1), Django REST framework
Database	PostgreSQL 17
Blockchain certification	Algorand (via LabTrace)
Web server	Nginx, Gunicorn
Deployment	AWS elastic beanstalk, EC2, S3, RDS
CI/CD	GitHub, AWS elastic beanstalk CLI

The frontend is developed using Bootstrap and JavaScript to ensure a responsive and intuitive interface optimized for multiple user roles (Administrators, Principal Investigators, and Project Members). The backend is powered by the Django web framework (Forcier et al., 2009), chosen for its scalability, built-in security features, and seamless integration with third-party services. Django handles all application logic, authentication workflows, and request-response lifecycles.

Integration with external platforms is achieved through dedicated APIs and service connectors. Overleaf integration is achieved through a custom-built API, enabling real-time synchronization and management of collaborative LaTeX documents directly within the platform. GitHub repositories are accessed using GitPython and RESTful API calls to enable version-controlled source code management. For dataset management, IntegriLAB incorporates DataLad through an in-house developed API, allowing users to link local or remote datasets and synchronize changes within their project workspace. LabTrace integration introduces blockchain-based certification. A custom SDK enables secure communication with the Algorand blockchain to timestamp and verify project milestones, ensuring tamper-proof records for key contributions.

All persistent data is stored in a PostgreSQL relational database, managed through Django’s Object-Relational Mapper (ORM). This abstracts complex SQL operations and streamlines data access. User roles and permissions are managed via Django’s authentication system, implementing a role-based access control (RBAC) model to enforce secure, hierarchical access to resources based on user roles.

IntegriLAB is deployed on the AWS cloud platform, ensuring scalability, reliability, and robust security, as depicted by the solid-line diagram in Figure 3. The system dynamically scales to meet traffic demands, running on Amazon EC2 instances with the PostgreSQL database hosted on Amazon RDS, which provides automated backups and failover management. Static assets, including templates, CSS files, research document logs, are stored securely in Amazon S3 (Simple Storage Service). Data transmission is secured using SSL/TLS encryption, and additional protection is provided through AWS Web Application Firewall (WAF) rules.

Although the current deployment utilizes AWS for operational convenience and scalability, the platform architecture remains portable and can be deployed on institutional servers or local infrastructure depending on resource availability. This allows research labs with limited infrastructure to run the framework while maintaining full functionality. In addition, because the platform primarily manages metadata rather than full datasets, its computational and storage requirements remain modest, helping to reduce operational costs. This flexibility enables scalable adoption and supports long-term sustainability across diverse research environments. The source code of the IntegriLAB application is available in a public Git repository for academic and research reference.^15^

### 3.4 Data security and privacy

IntegriLAB incorporates a robust security framework designed to protect user data and enforce strict access controls. The platform follows GDPR-aligned practices by minimizing the storage of personal information. Only essential metadata, such as timestamps, authorship information, and file names is retained, while no substantive research data or sensitive content is stored on the system. This approach significantly reduces the risk of data exposure and simplifies regulatory compliance. Only minimal user information is required to create an account and configure integrated tools.

All data is encrypted both in transit and at rest, using SSL/TLS protocols for secure communication and Amazon RDS encryption^16^ for database storage. Role-Based Access Control (RBAC) defines user permissions, ensuring that users such as Administrators, Principal Investigators, and Members can access only the resources appropriate to their role. User authentication is managed through Django’s built-in mechanisms, providing secure registration, login, and password management. Furthermore, AWS Web Application Firewall (WAF) is configured to defend the platform against common web threats, strengthening the overall security posture.

### 3.5 System validation

Comprehensive testing of IntegriLAB was conducted across multiple levels to ensure functionality, reliability, usability, and generalizability. The platform is designed to integrate widely used, platform-independent tools (DataLad, GitHub, Overleaf) to support research workflows by tracking datasets, associated analyses, and reported findings, independent of workflow scale or complexity. While the current study demonstrates a single neuroimaging project, the design allows deployment across different laboratories, datasets, and user profiles with minimal workflow changes.

Functional testing validated core operations, including project creation, user registration, repository setup, and blockchain certification. It confirmed that Principal Investigators (PIs) could approve team members and configure repositories, while team members were able to commit changes and request certification. Integration testing ensured seamless data exchange between IntegriLAB and external platforms, verifying that updates in any system were accurately reflected across all others.

Performance testing assessed the platform’s responsiveness under varying loads, including projects with multiple users, frequent commits, and large datasets. As it primarily tracks metadata from connected repositories rather than handling full datasets or documents, ensuring lightweight operation for typical small-to-moderate projects. The platform focuses only on the most recent updates in each repository rather than traversing the entire commit history which ensures that synchronization and logging remain efficient even as the number of commits, documents, or datasets grows. However, for very large Overleaf repositories with many documents or images, fetching the latest updates may take longer, resulting in minor delays in metadata synchronization. The latency for generating certification through LabTrace is negligible, as blockchain timestamps are applied only to key milestones.

Security testing verified robust authentication and authorization across user roles and assessed the integrity of blockchain-based certification. User Acceptance Testing (UAT) involved real project teams operating in a controlled environment, evaluating usability, workflow alignment, and user satisfaction. Feedback from UAT informed iterative improvements to the platform’s design and interface.

## 4 Case study: enhancing research transparency in schizophrenia study using IntegriLAB

**FIGURE 5:**
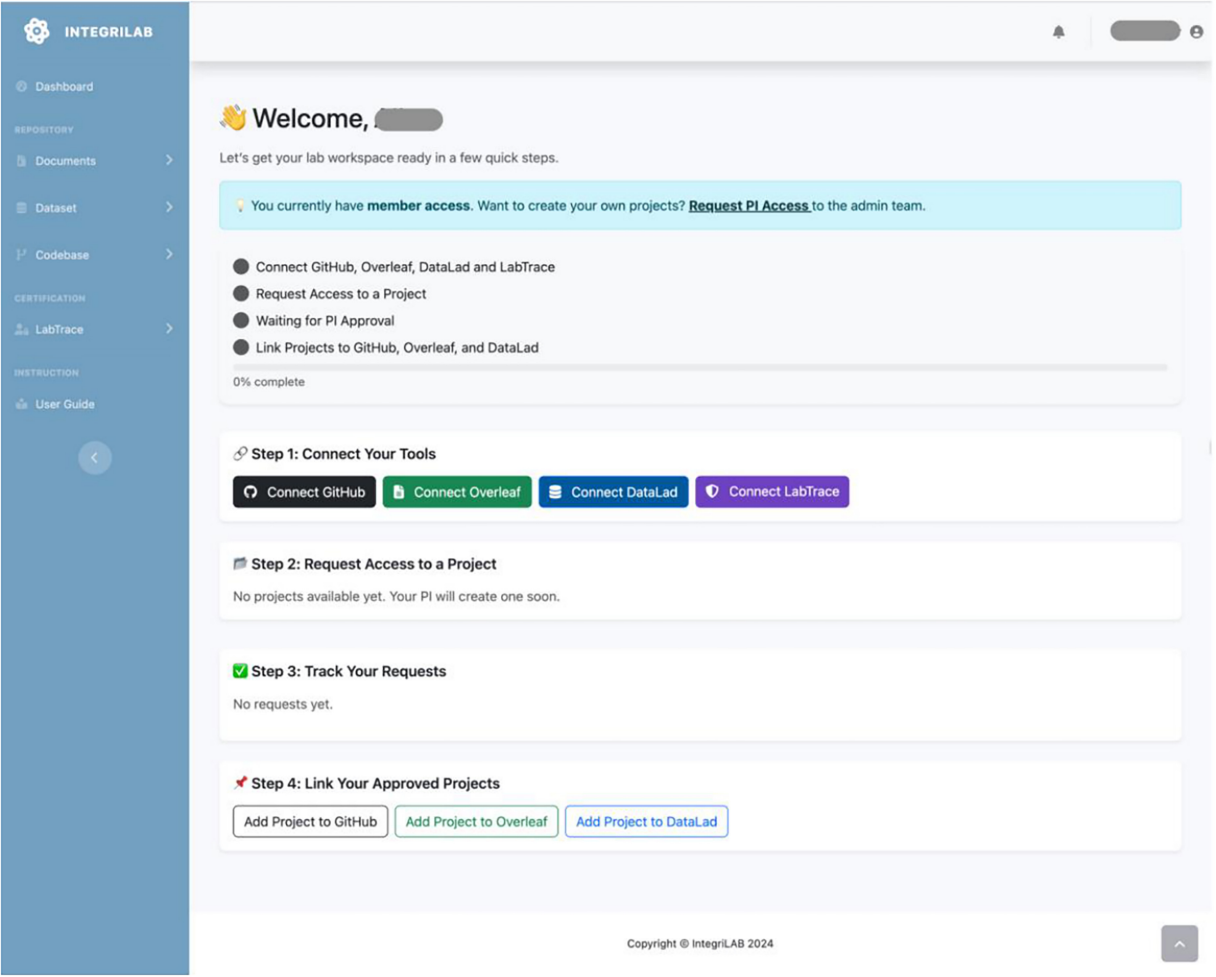
IntegriLAB dashboard showing guidance for new users, including steps to request project access, connect with relevant tools, and link the project to associated repositories.

IntegriLAB was applied in a schizophrenia (SCZ) research project investigating dopamine synthesis capacity using [18F] FDOPA PET imaging and radiomics to predict antipsychotic treatment response (Schiulaz et al., 2025). This study involved complex workflows spanning data storage, code analysis, and documentation. As illustrated in Figure 5, the IntegriLAB dashboard provides step-by-step guidance for new users. Project initiation requires registration and administrative approval for the Principal Investigator (PI), after which project members can begin integrating and managing resources. In this study, the team linked IntegriLAB with DataLad for version-controlled imaging and feature datasets (CSV format), GitHub for Python and MATLAB analysis scripts, and Overleaf for collaborative report writing.

**FIGURE 6:**
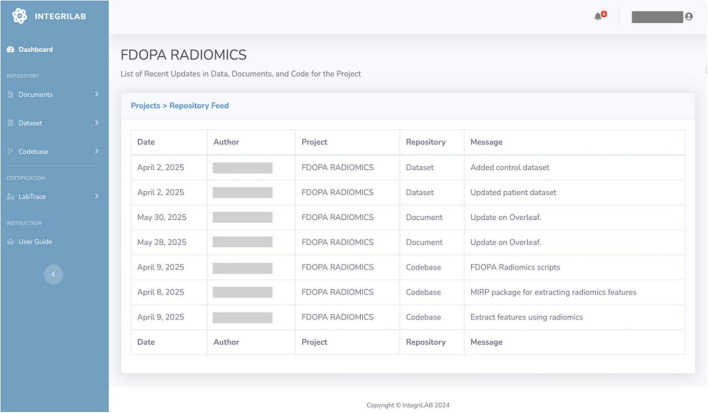
IntegriLAB interface displaying tracking of data, document and code changes, including timestamps, authorship, and descriptive messages, to ensure transparency and traceability in the schizophrenia FDOPA Radiomics study.

When work reaches a stage suitable for team review or reporting, users log metadata into IntegriLAB, including authorship, timestamps, and workflow transitions. It’s an automated process as the project is already connected with the respective repositories. This creates a timeline that the team can reference during collaboration. Figure 6 shows the interface, highlighting ongoing tasks along with detail change messages from different research tools, author details, and time logs for updates across repositories.

**FIGURE 7:**
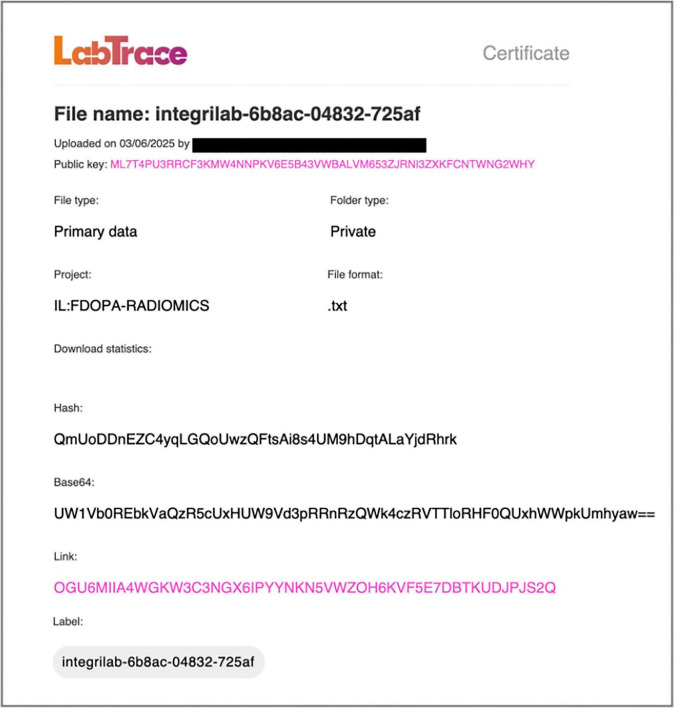
Sample digital certificate generated by LabTrace via IntegriLAB, displaying project and file metadata, author identity verified through a public key, and a cryptographic fingerprint ensuring data integrity. The access link directs to the certificate’s metadata location on the Algorand blockchain.

Blockchain certification is applied when a research milestone or result needs to be formally recorded. At that point, the system certifies that a specific version of the analysis code from GitHub, when executed on the corresponding dataset from DataLad, produces the result documented in Overleaf. An example certificate is shown in Figure 7. Each certificate records project information, author details, and the file’s cryptographic fingerprint (hash and Base64 encoding), establishing provenance and ownership through public key infrastructure. It also includes an immutable access token linking to the certification record stored on the Algorand blockchain.

Overall, integrating IntegriLAB required minimal workflow changes, as it connects with existing tools rather than replacing them. The platform offers clear advantages, including integrated tracking of datasets, code, and documents, automated logging of contributions, and tamper-proof certification of key milestones. Limitations include the need for users to ensure repositories are correctly connected with the application, some expertise in using GitHub, DataLad, and Overleaf, and a small processing delay during blockchain certification; however, this delay is negligible and does not interfere with routine analysis workflows. Despite these considerations, the platform provides a centralized interface for managing the complex workflows of neuroimaging studies.

## 5 Discussion

IntegriLAB was developed as a proof-of-concept platform to integrate data, document, and code management within a unified, web-based environment. By linking existing research tools, DataLad for data versioning, Overleaf for collaborative documentation, and GitHub for code management; IntegriLAB facilitates end-to-end traceability of neuroimaging workflows. IntegriLAB extends versioning and documentation principles across the broader research lifecycle by maintaining detailed logs of research activities, allowing team members to monitor progress, revisit prior states, and restore earlier versions when necessary. Importantly, the platform does not require the transfer or central storage of sensitive datasets, scripts, or documents. Instead, it aggregates and certifies metadata and activity logs from distributed systems, enabling researchers to continue using their preferred environments while achieving centralized oversight and coordination. The addition of LabTrace, a lightweight blockchain-based verification layer, ensures cryptographic certification of research activities while maintaining low computational overhead.

Traditional ELNs have primarily focused on improving project organization and data documentation. However, most existing systems lack interoperability across platforms, hindering seamless integration and communication between data, analysis, and documentation systems. As a result, establishing verifiable linkages across these components often requires manual intervention, increasing the risk of human error and compromising workflow reliability. Tools such as LabArchives, JupyterLab, Benchling etc., offer partial solutions but do not provide immutable certification of research actions. In contrast, IntegriLAB’s integration of blockchain-based verification introduces a transparent audit trail that enhances reproducibility and accountability without disrupting researchers’ established workflows.

Reproducibility in neuroimaging research often suffers due to fragmented data management and inconsistent record keeping. IntegriLAB directly addresses these issues by combining structured data handling with verifiable authorship and provenance tracking. This framework not only improves transparency in collaborative environments but also provides a mechanism for institutions and journals to validate research outputs, fostering greater confidence in published findings.

While the integration of DataLad, Overleaf, and GitHub demonstrates the system’s feasibility, the current implementation relies on user familiarity with version-controlled environments. Broader adoption may require additional user interface simplifications and automated synchronization features. Incorporating real-time collaboration tools for task management and progress tracking could further enhance team coordination. To assess scalability and real-world applicability, future case studies will focus on integrating IntegriLAB with large-scale data-sharing initiatives such as Psy-ShareD (Allen et al., 2025), enabling validation across diverse, multi-institutional research settings.

## 6 Conclusion and future work

Scientific research increasingly relies on the coordinated management of interdependent components such as code, data, computational environments, and documentation. While platforms like GitHub and version control systems have improved collaborative software development, other aspects of the research workflow remain fragmented and difficult to manage. IntegriLAB represents a significant step toward a transparent, verifiable, and sustainable research infrastructure in neuroimaging. By bridging the gap between data management and research integrity, it advances the broader vision of open, accountable, and reproducible science. Future development will focus on enhancing interoperability with widely used documentation platforms such as Microsoft Word and Google Docs, allowing researchers to work within familiar environments while maintaining synchronized activity logs. Integrating Docker technology could further strengthen reproducibility by enabling complete replication of computational environments, including software dependencies and configurations.

## Data Availability

The original contributions presented in the study are included in the article/supplementary material, further inquiries can be directed to the corresponding author/s.

## Footnotes

1
https://www.labarchives.com/

2
https://www.scinote.net/blog/electronic-lab-notebook-guide/

3
https://www.benchling.com/

4
https://www.elabnext.com/

5
https://genemod.net/

6
https://github.com/

7
https://www.overleaf.com/

8
https://labtrace.io/

9
https://research-and-innovation.ec.europa.eu/strategy/strategy-research-and-innovation/our-digital-future/open-science_en

10
https://www.scispot.com/blog/labarchives-pricing-guide-plans-costs-features-explained?utm_campaign=xyz123

11
https://en.wikipedia.org/wiki/Blockchain

12
https://www.iberdrola.com/sustainability/green-cryptocurrencies

13
https://solartechonline.com/blog/most-energy-efficient-cryptocurrency-guide-2025/

14
https://docs.github.com/en/repositories/working-with-files/managing-large-files/about-large-files-on-github

15
https://github.com/Rubaida001/integriLAB01.git

16
https://docs.aws.amazon.com/AmazonRDS/latest/UserGuide/Overview.Encryption.html
